# Modern Approaches to Testing Drug Sensitivity of Patients’ Tumors (Review)

**DOI:** 10.17691/stm2020.12.4.11

**Published:** 2020-08-27

**Authors:** I.N. Druzhkova, M.V. Shirmanova, D.S. Kuznetsova, М.М. Lukina, Е.V. Zagaynova

**Affiliations:** Junior Researcher, Fluorescent Bio-imaging Laboratory, Research Institute of Experimental Oncology and Biomedical Technologies; Privolzhsky Research Medical University, 10/1 Minin and Pozharsky Square, Nizhny Novgorod, 603005, Russia;; Deputy Director for Science, Research Institute of Experimental Oncology and Biomedical Technologies; Privolzhsky Research Medical University, 10/1 Minin and Pozharsky Square, Nizhny Novgorod, 603005, Russia; Head of Fluorescent Bio-imaging Laboratory, Research Institute of Experimental Oncology and Biomedical Technologies; Privolzhsky Research Medical University, 10/1 Minin and Pozharsky Square, Nizhny Novgorod, 603005, Russia;; Researcher, Regenerative Medicine Laboratory, Research Institute of Experimental Oncology and Biomedical Technologies; Privolzhsky Research Medical University, 10/1 Minin and Pozharsky Square, Nizhny Novgorod, 603005, Russia;; Junior Researcher, Fluorescent Bio-imaging Laboratory, Research Institute of Experimental Oncology and Biomedical Technologies; Privolzhsky Research Medical University, 10/1 Minin and Pozharsky Square, Nizhny Novgorod, 603005, Russia;; Corresponding Member of Russian Academy of Sciences, Rector; National Research Lobachevsky State University of Nizhni Novgorod, 23 Prospekt Gagarina, Nizhny Novgorod, 603950, Russia Chief Researcher, Laboratory of Optical Coherence Tomography, Research Institute of Experimental Oncology and Biomedical Technologies Privolzhsky Research Medical University, 10/1 Minin and Pozharsky Square, Nizhny Novgorod, 603005, Russia;

**Keywords:** medical oncology, individualized cancer therapy, cancer treatment selection, molecular genetic tumor testing, drug testing *in vitro*.

## Abstract

Drug therapy is still one of the basic techniques used to treat cancers of different etiology. However, tumor resistance to drugs is a pressing problem limiting drug treatment efficacy. It is obvious for both modern fundamental and clinical oncology that there is the need for an individual approach to treating cancer taking into account the biological properties of a tumor when prescribing chemo- and targeted therapy. One of the promising strategies is to increase the antitumor therapy efficacy by developing predictive tests, which enable to evaluate the sensitivity of a particular tumor to a specific drug or a drug combination before the treatment initiation and, thus, make individual therapy selection possible.

The present review considers the main approaches to drug sensitivity assessment of patients’ tumors: molecular genetic profiling of tumor cells, and direct efficiency testing of the drugs on tumor cells isolated from surgical or biopsy material. There were analyzed the key directions in research and clinical studies such as: the search for predictive molecular markers, the development of methods to maintain tumor cells or tissue sections viable, i.e. in a condition maximum close to their physiological state, the development of high throughput systems to assess therapy efficiency. Special attention was given to a patient-centered approach to drug therapy in colorectal cancer.

## Introduction

Substantial advance in science in comprehending carcinogenesis mechanisms has resulted in an established opinion among oncologists that cancer therapy should be individualized. Due to the effect of numerous factors providing intra- and inter-tumor heterogeneity and high adaptive capacity of cancer cells, there are different responses of tumor cells of the same type and stage to a similar drug therapy in different patients. As a final result, it leads to insufficient therapy efficacy, side effects development, and unreasonable expenses.

The first attempts to assess the tumor cells sensitivity of a certain patient to drugs in order to choose the most effective drug therapy were as early as in 1970–80-s. However, they were not introduced into clinical practice due to a number of problems. In particular, long-term culture of patients’ tumor cells as cell cultures was noted to change their condition. Secondly, the cultured cancer cells can respond to chemotherapeutic drugs differently than those in a patient body. And, moreover, the post-treatment analysis of cell condition required the involvement of highly-qualified pathologists.

In the past decade the problem of patient-centered drug therapy got a new lease of life. Due to the technological development in molecular and cellular biology, as well as the broadening of methods range used to study structural and functional state of cells and tissues, there is the feasibility to comprehensively and relatively quickly investigate postoperative and biopsy material *in vitro*. Some research groups are developing the techniques for cancer cell isolation from solid tumors, maintenance of cell and tissue samples, and suggesting cultural 3D systems to establish the conditions maximum close to physiological conditions, and modeling the relationship of tumor cells and their natural microenvironment. Other research groups are concentrating their efforts on maximum informative ways to assess a therapeutic response of tumor cells. Others are searching for molecular markers for expected therapy efficiency. However, each specific tumor site requires a peculiar unique protocol, and it is due to very different biological properties of cells of different histogenesis.

According to cancer morbidity structure, colorectal cancer is one of the most common worldwide, it ranks third in men and second — in women. In Russia, colorectal cancer accounts for over 11% of all cancers. Conventional chemotherapy is considered the basic technique in medical oncology of colorectal cancer. Targeted therapy in colorectal cancer is used only if there are metastases and there are no certain mutations. However, the choice of target agents for colorectal cancer therapy is limited, and their efficiency is compelling. Despite the availability of operative material, drug sensitivity of colorectal tumors is under-investigated so far.

The present review is devoted to the analysis of a worldwide trend in developing the techniques to test the drug sensitivity of patients’ tumors. The study systemizes the general data on medical oncology, describes the current approaches to the assessment of tumor sensitivity to chemo- and targeted therapy, as well as the basic evaluation techniques of tumor cell responses to therapeutic treatment. Special attention was paid to the implementation of a patient-centered approach in colorectal cancer therapy.

## Medical oncology and grounding for the necessity for treatment individualization

Currently, the main cancer treatment techniques are surgery, radiotherapy, and drug therapy including hormone-, chemo-, and targeted therapy.

The drug therapy selection is based on classical clinical diagnostic criteria such as: a tumor size, a histological analysis, as well as the presence or absence of standard markers in case of targeted therapy.

Chemotherapy is a standard technique to treat tumors of various localizations, and based on chemotherapeutic agents administered to a patient [1]. So far, there are several different groups of antitumor agents with different mechanisms of action:

1. alkylating antineoplastic agents — are aimed at damaging DNA molecules;
2. metabolic antagonists — inhibit a number of important biochemical processes necessary for proliferative cell function, and they result in apoptosis activation;
3. anthracycline antibiotics — inhibit DNA molecule synthesis and affect cell membrane permeability;
4. topoisomerase inhibitors — selectively damage MNA molecule structure and tumor-cell division at different mitosis stages;
5. mitotic inhibitors — inhibit mitosis and cell division.

In clinical protocols of chemotherapy, antitumor agents are used either in a combination with each other or as a mono-agent pre- and postoperatively. Therapy regimen selection depends on a tumor site, the cancer stage, and other characteristics of clinical presentation [2, 3].

According to the recommendations [4, 5], adjuvant colorectal cancer therapy includes the administration of the following agents: oxaliplatin and 5-fluorouracil (regimens: FOLFOX, FLOX) or capecitabine (XELOX regimen). Drug therapy of metastatic colorectal cancer in case of resectable metastases the same agents and regimens are recommended, as well as mono-therapy by fluoropyrimidines; in irresectable metastases — Irinotecan (FOLFOXIRI regimen) is added to oxaliplatin and 5-fluorouracil.

The advent of targeted therapy has significantly changed an approach to cancer treatment enabling to administer agents relying on tumor characteristics of a particular patient, and showing the possibility of patient-centered approach [6]. Currently, nine prognostic markers have been introduced into clinical practice, which enable to determine the sensitivity to specific treatment and administer target agents [7]. The main types of target agents are small molecules — inhibitors of tyrosine kinase and serine/threonine kinases and monoclonal antibodies to HER2/Neu receptors, epidermal growth factor receptor (EGFR) and vascular endothelial growth factor (VEGF).

In colorectal cancer therapy, appropriate target agents are administered relying on mutation analysis. According to medical oncology of rectal carcinoma, colon cancer, and recto-sigmoid junction [4, 5], tumor molecular profile should be taken into consideration when administering targeted therapy and choosing a target agent. In case of the lack of mutations in *KRAS* and *BRAF* genes, anti-EGFR-agents: Cetuximab or Panitumumab — are indicated. However, targeted therapy is not a basic technique in colorectal cancer, and administered in metastatic cancer only.

Despite an increased understanding of malignant cell transformation, the efficiency of most cancers is still low. One of the causes of drug therapy failure is tumor heterogeneity: a complex of characteristics presenting inter- and/or intra-tumor differences. A tumor is a complex system, heterogeneous by its cellular space, a molecular profile, architecture, and spacious organization. Phenotypic, genetic, epigenetic, and other characteristics are congenial for some cells and cell populations forming an extremely complicated and heterogeneous structure [8, 9]. Tumor heterogeneity is a necessary condition for cancer progressing, tumor cells surviving in unfavorable conditions, including the effect of anti-tumor agents and drug resistance development [10].

Multidrug resistance is a well known phenomenon depending on a number of nonspecific factors including high tumor plasticity and heterogeneity, and secondary genetic damages tumor cells acquire, tumor microenvironment [11, 12]. Drug resistance can be inherited (it is also called pre-existing or initial) and acquainted (or adaptive) arising under therapy pressure. Not infrequently, chemotherapeutic agents, which are effective for a primary tumor site, appear to fail in metastases or in recurrent tumors. The intensity of universal resistance mechanisms should be revealed before treatment, if possible.

Both: classical cytotoxic chemotherapy and targeted chemotherapy are accompanied by a number of side effects. Marked side effects require drug correction. They decrease life quality and sometimes can result in therapy cessation.

All the above-mentioned reasons have led to a new insight into cancer therapy, and indicate clearly the necessity for patient-centered medicine consisting in an elaborate study of patient tumor material and the selection of drugs with maximum efficiency for a particular tumor [13].

## Molecular genetic analysis to implement an individual approach

Molecular factors, in particular, the presence of mutations in *KRAS*, *NRAS*, and *BRAF* genes associated with certain histological tumor type can be a significant prognostic criterion and determine initial or acquired sensitivity of tumor cells to some forms of treatment including radiotherapy, many types of cytostatics, some target agents, gene therapy, and certain techniques of immune therapy [14, 15]. These genes are key protooncogenes activated in most malignancies including colon cancer. They encode RAS proteins, which are the first members of a cascade of kinases leading to the activation of signal paths and gene transcription regulating cell differentiation and proliferation. The database of the Catalogue of Somatic Mutations in Cancer (https://cancer.sanger.ac.uk/cosmic) shows that about 34% of colon tumor samples analyzed have *KRAS* mutations, 10% — *BRAF*, and 4% — *NRAS*.

Molecular genetic analysis of mutation status of RAS-cascade of *KRAS*, *NRAS*, and *BRAF* genes are of great prognostic and predictive importance in colorectal cancer therapy. Main mutations in RAS genes in colon tumors concentrate in exon 2, codons 12 and 13. However, there can be mutations in exon 3, codon 61, as well as in exon 4, codons 117 and 146. Mutation status of codons 12 and 13 of *KRAS* gene is the most familiar biomarker in targeted anti-EGFR-therapy of patients with metastatic colorectal cancer [16]. *KRAS* activation due to mutation has been proved to nullify the effect of EGFR inhibition by monoclonal antibodies. Thus, the presence of mutant alleles of *KRAS* gene is an independent predictive marker of the efficiency of EGFR inhibitors therapy [17]. Mutations affecting codon 61 damage hydrogen bonds between *RAS* and protein-inactivators result in the same effect that there is in codon 12 and codon 13 damages. Codon 146 mutations are not accompanied by significant changes of the protein activity. However, these mutations have a negative effect resulted from the accumulation of a defective protein against the background of allelic imbalance — increased abundance of a mutant gene or its transition in homozygous state. A number of clinical trials showed that patients with a wild type of *KRAS* and *NRAS* genes in a tumor would get the most out of antibody therapy combined with standard chemotherapy compared to patients without *KRAS* gene mutation in exon 2 [18–20].

*BRAF* gene encodes intracellular protein, which is a component of RAS–MAPK and RAS–MEK–ERK signal cascades regulating cell proliferation in response to external mitogenic stimuli. The most frequent activating mutation of *BRAF* gene is single nucleotide substitution, which affects codon 600 of exon 15 — V600E in 95% cases. There are conflicting data on a predictive role of *BRAF* V600E mutation in regard to a tumor response to anti-EGFR-therapy, and prognostic significance of disease progression [21, 22]; however, patients with *BRAF* gene mutation in a tumor are known to be a separate group with an unfavorable clinical course. In addition, a prognosis for patients with metastases and a mutation in *BRAF* gene is extremely unfavorable due to aggressive tumor growth. However, determining *BRAF* gene status along with *KRAS* will enable to correctly select patients for therapy by anti-EGFR-monoclonal antibodies. Combined use of inhibitors of *EGFR*, *BRAF*, *MEK* genes shows promising results, and the introduction of one more biomarker along with *KRAS* and *NRAS* genes will enable to enhance a patient-centered approach in colon cancer therapy [23].

Colon cancer carcinogenesis is characterized by mutation accumulated in genes controlling the growth and differentiation of epithelial cells resulting in their genetic instability [24]. One of such genetic alterations is microsatellite instability, which is characterized by an impaired repair mechanism of unpaired DNA bases. It leads to the fact that mutations in a cell genome are accumulating at higher speed than normal. Microsatellite instability occurs in 15% sporadic colon tumors, and in all cases of Lynch syndrome. Impairments in DNA system repair result in insertions and/or deletions of nucleotide repeats in DNA. It is possible to reveal failed repairability of unpaired DNA bases by DNA microsatellite length [25]. There has been found the relation between *BRAF* gene mutation and the repair system state of unpaired DNA bases. In microsatellite instability, *BRAF* gene mutation frequency reaches 50%, while in microsatellite stable tumors — the gene mutations occur rarely. In addition, only in the latter case mutations in *BRAF* gene are associated with low survival rate at early stages of the disease [23, 26]. The marker is more used for disease prognosis rather than a ground for choosing some therapy.

It should be noted that in case of targeted therapy, a preliminary analysis enables to determine a target but not take into account the tumor sensitivity or resistance degree. For example, even patients with no mutations in *KRAS* and *NRAS* genes were found [27, 28] to have a therapeutic response to anti-EGFR-agents only in 20–30% cases, and when combined with chemotherapy — 65–70%.

For tumor response prognosis to usual chemotherapeutical agents, there is also used an approach based on the cell genome and proteome analysis. In particular, some markers are known to be used to prognosticate the efficiency of agents widely applied in colorectal cancer: 5-fluorouracil, Irinotecan, and Oxaliplatin.

Tolerance and efficiency of fluoropyrimidines largely depend on their systemic and intra-tumor metabolism. A key enzyme of 5-fluorouracil breakdown is dihydropyrimidine dehydrogenase (DPD). Some individuals have a hereditary defect, due to which both (paternal and maternal) *DPD* gene replicas fail to produce a normal protein. Such people accounting for about 0.1% population are characterized by marked intolerance to fluoropyrimidines: even the first administration of a standard dose of 5-fluorouracil can result in fatality. Detection of people with systemic DPD inactivation requires a complete sequencing of a corresponding gene [29].

Another parameter influencing the outcome of the treatment by 5-fluorouracil and its derivatives is intra-tumor DPD activity. If systemic DPD deficiency determined by inherited mutation in the gene is of serious hazard, then low DPD activity in tumor tissue itself contributes to the agent accumulation within the mass lesion. Many tumors have reduced DPD expression compared to normal tissues — it is the peculiarity of carcinomas that creates a certain therapeutic window for fluoropyrimidines. Numerous studies have shown colorectal cancer with low DPD to demonstrate a more prominent response to 5-fluorouracil therapy [30].

Another molecular factor associated with colorectal cancer sensitivity to 5-fluorouracil is thymidylate synthase (TS). The enzyme is considered the main target of 5-fluorouracil. High intra-tumor TS expression is frequently associated with tumor resistance to fluoropyrimidines. It can be explained by the fact that a therapeutic concentration of 5-fluorouracil appears to be insufficient to bind an excess amount of TS molecules [31].

Thymidine phosphorylase (TP) is a key enzyme of synthesis and degradation of pyrimidine nucleotides. Anti-apoptotic and angiogenic effects of TP are involved in colorectal cancer growth and metastasing. Moreover, TP is a key enzyme to activate prodrugs of 5-deoxy-5-fluorouridine into 5-fluorouracil [32]. TP hyper-expression is related to a bad prognosis due to an increased infiltrating capacity, more active growth, and metastases. However, TP expression is necessary to provide a curative effect of 5-fluorouracil. Thus, regardless of the fact that TP is a marker of an unfavorable course of the disease and tumor angiogenic potential, it also serves as a marker for anti-angiogenic agents, and is a 5-fluorouracil activator [33].

Generally, over the last years, the development of this sphere of clinical oncology has somewhat ceased. Firstly, 5-fluorouracil and its derivatives have been used rarely as a monotherapy, and correspondingly, when analyzing a tumor response to a combination of drugs it is cumbersome to reveal which component of a treatment schedule has contributed to treatment success. Secondly, most researchers prefer to use the easiest and readily available technique to determine the expression of DPD, TS, and other molecules — immunohistochemistry, which is notable for poor intermediate precision due to the variety of antibodies used, and subjectivity when assessing staining intensity [29].

Irinotecan — topoisomerase I inhibitor — at the time made a considerable contribution to effective colorectal cancer therapy; however, it showed significant population variability in regard to the therapy tolerance. Sub-studies revealed that one of the main parameters determining the intensity of side effects in Irinotecan administration is *UGT1A1* gene polymorphism. The gene is characterized by population diversity concerning the number of dinucleotide repeats of thymidine adenine in promoter (regulatory) gene region. The overwhelming majority of researchers agree that the presence of *UGT1A1* gene allelic variants are associated with high toxicity of Irinotecan. There are few research works dealing with studying sensitivity determinants of colon cancer to Irinotecan rather than the analysis of Irinotecan tolerance [34]. In particular, a large variety of preclinical studies and clinical trials indicate that the response probability to Irinotecan can be associated with intra-tumor expression of its target — topoisomerase I. Unfortunately, few studies and dissimilarity of the techniques used to determine topoisomerase I status prevent from making final conclusions on the issue [29].

Oxaliplatin by its efficiency is comparable with Irinotecan, and in most cases it can be its alternative in therapy planning. In Russia, Oxaliplatin is used on a somewhat more frequent basis than Irinotecan — such choice of patients and doctors is related to a lower risk of alopecia and severe diarrhea. However, the choice between Oxaliplatin and Irinotecan is a spectacular example of clinical settings when an analysis of a predictive marker could be a decisive component in determining the disease management.

A considerable number of articles are concerned with ERCC1 (DNA repair enzyme) expression status application prospects. Low ERCC1 is considered to be associated with higher probability of a response to therapy, since the enzyme can participate in repairing DNA-adducts formed as a result of platinum-containing agents [35]. Nevertheless, the researchers in this field face the same difficulties as those studying the use of fluoropyrimidines [36].

A detailed analysis of gene expression enables to develop test-systems to prognosticate a clinical course and give grounds for drug choice. For example, there are several commercial test-systems used in breast cancer therapy: Oncotype DX (Genomic Health, USA), Prosigna (PAM 50, NanoString Technologies, USA), EndoPredict (Myriad Genetics, USA), and MammaPrint (Agendia, Netherlands). These test-systems are developed for early stages (I, II stages) and are primarily for hormone-positive tumors. From 21 to 70 genes can be analyzed using test-systems, they showing a tumor grade, the presence of hormones receptors and targets for prescribing targeted therapy [37]. However, the significance of such researches is still equivocal, since most patients have an intermediate risk (judging by a risk evaluation scale) that is unilluminating when choosing and grounding treatment; clinical findings are also ambiguous. However, similar test-systems are being developed for other cancer types including colorectal carcinoma. Currently, there has been studied clinical significance of the main genes involved in colorectal cancer carcinogenesis [38, 39].

## Main approaches to testing drugs on patients’ tumor cells

One of the first approaches to individual therapy selection was that one based on the treatment results of laboratory animals with patient-derived xenografts (PDX). The technology was first described as early as in 1969 [40]. Its backbone is in the following: small tumor fragments derived from patients intra-operatively are transplanted to immunodeficient mice. Tumors grown in such mice are then re-engrafted to similar immunodeficient mice-recipients, which are treated by a certain chemotherapeutic agent. A therapeutic response is assessed by a standard technique — by tumor growth inhibition. It is important that PDX models, as a rule, preserve molecular characteristics, cellular and pathomorphological structure of initial patient tumors [41–43]. Moreover, a cytogenetic analysis of tumor cells isolated from PDX shows substantial similarity of a genetic profile and genes expression profile in PDX and initial patient tumors [44–46]. PDX models were taken for different solid tumor types. PDX drug response was proved to correlate well with a clinical response in patients [47–50]. The assessment of approximately 300 cases for 13 tumor types showed a good correlation between a patient’s response and PDX therapeutic response — from 70 to 100%.

Although PDX models have distinct advantages, there are some limitations, which prevent from using them widely in personalizied medicine. For example, for tumor xenograft survival, a very long period of time is required, about 4–8 months [51–53], and some extra time to create daughter tumor xenografts in order to test therapeutic regimens on mice. In addition, PDX grafting frequency in mice for most cancer types usually does not exceed 50%, and for breast cancer, prostate cancer, and renal cell carcinoma the percentage is significantly lower [54–56]. Highly immunodeficient mice themselves are expensive and require specific clean housing conditions, and highly qualified staff. So, despite relative success of the technique, it is one of the most costly, labor-consuming, and has a long runtime that makes it unacceptable to be used in practice [57].

The specified situation determines an urgent need in rapid and safe alternative methods to assess patients’ tumor sensitivity to drugs. In this problem, great attention is given to the development of techniques used to determine the chemosensitivity of tumor cells on *in vitro* material isolated from tumors.

Early passage lines taken from patient’s tumor are known to present better tumor properties than commercial cell lines, and therefore, they can predict accurately the chemosensitivity of a particular tumor [58]. To derive cancer cell cultures from a tumor is an intricate problem due to frequent contamination of primary material, and more rapid growth of stromal cells compared to tumor ones [59, 60]. Currently, the success in cancer cells isolation from most solid tumors is achieved only in 10–40% cases [60, 61].

Two main ways of taking temporary tumor cultures are the direct cancer cells isolation from tumor tissues (tissue organoids or cell suspension) and a xenograft technique, when an animal organism is a primary recipient of tumor cells [60, 62]. The major shortfall of the latter is an undesirable selection of tumor cells in an animal body, while the information on chemosensitivity for such cells can be much different from initial population. In this regard, the most adequate assessment method for primary chemosensitivity is the direct cancer cells isolation from tumor cells.

According to a direct cancer cells isolation technique, culture material is taken observing aseptic conditions by dissecting appropriate tumor fragments paying attention to the viability of cell elements. Culture tissue should have no necrotic areas, be sterile and abundant in the cells which are to be cultured. Tissue fragments are cut into small pieces, 1–3 mm in diameter in size, and put in a culture medium [60]. One of the variants is to derive, wherever possible, homogeneous cellular suspensions from tumor tissue samples [62]. Recently, one or several enzymes (trypsin, liberase, collagenase) are used to derive cellular suspensions, it depends on a tissue type. Cellular suspension portions collected are washed to free from enzymes, and centrifuged in ficoll gradient to free from associated cell fractions. The cells purified in this way are resuspended in a culture medium and transferred into appropriate dishes in accurately measured amounts [62]. The technique enables to derive living cell masses free from stroma. Then, obtained tumor cells are cultured in culture flasks using a standard procedure (37ºС, 5% СО_2_, moist atmosphere). To analyze certain characteristics, tumor cells are disseminated in culture dishes or plates.

Intercellular substance is also relevant in tumor growth and its chemotherapeutic resistance. Primarily, it is collagen, as well as laminin and fibronectin. For instance, it has been shown that cell survival rate when exposed to such agents as Cisplatin, 5-fluorouracil, and Epirubicin, and when performing researches on decellularized tumor stroma is 20–60% higher than on plastics [63]. Therefore, extracellular matrix, e.g., collagen, is introduced in test-systems to determine chemosensitivity of tumor cells. Since the 1990-s, there has been developed a new *in vitro* chemosensitivity test using collagen gel droplet embedded culture [64]. The method complements a 3D tumor model using collagen as an intercellular substance. When applying a collagen gel droplet embedded culture technique, one can assess an increase/decrease of tumor spheroid size in reference to control when exposed to chemotherapeutic agents by a series of luminal images taken by a microscope [64, 65]. Currently, the technique is undergoing validation, including that for colorectal cancer [65].

It is commonly known that a tumor has complex heterogeneous structure, and consists of different-type cells, which intercommunicate and interact with tumor microenvironment. Stromal cells are active participants of carcinogenesis, and contribute to the formation and manifestation of tumor distinguishing features, as well as take part in chemotherapy resistance developed in tumor cells [66]. Therefore, an important task for personified screening is drug sensitivity analysis not only on cell cultures, but also on more complex 3D models *in vitro* containing cells of different types. As 3D cultures the following ones are considered:

1. Tumor organoids, which are 3D tumor cell cultures; patient-derived tumor cells being cultured as spheroids. Organoids present cell-to-cell cooperation, as well as the interaction of cells and extracellular matrix. High productive drug testing (screening) methods based on organoids are suggested, they would predict a patient tumor response to therapy [67–70].

2. Tumor tissues disintegrated by micro-dissection and maintained in cultural conditions. In this case, tissue preparation includes bioptate mechanic fragmentation, which, however, can cause local tissue damage, though preserving an immunological profile [71, 72].

3. Organotypic slices of tumor tissue kept in cultural conditions. Slices are sections or of tumor tissue samples, 300–500 μm thick, from the primary tumor and placed into a culture medium [73]. Cultured slices present well tumor micro-environment; the method used to derive them is rather easy and not time-consuming, and can be applied in most solid tumors [74–76]. When cultured up to 7 days, slices have been found to preserve tumor morphological properties [74]. For breast cancer and pancreatic carcinoma there has been demonstrated the correlation of treatment results and drug testing on patient tumor slices [73, 77]. Recently, there has been achieved success in studying a drug effect on tumor slices derived from patient tumor xenografts grown on laboratory animals [78].

The analysis of 3D *in vitro* tumor models shows that their major problem is short-term maintenance in culture due to diffuse nutritional type, no vascularization, no circulation of substances; there are necroses and hypoxia in central 3D structure. Moreover, currently, there are no standardized systems with optimally matched culture conditions, using which it could be possible to perform high producing screening of anti-tumor drugs on a large scale. Microfluid systems exhibit high potential in solving such problems, since they enable 3D tissue models gain efficiency.

Microfluid systems, or chips, are devices to culture cells and tissues, and consist of optically transparent plastic, glass, and flexible polymers, e.g., polydimethylsiloxane (PDMS), with hollow chambers connected with a canal and pump system for perfusion, control and maintaining specified micro-environment conditions [79]. The systems got their name ‘chips’ due to a manufacturing technology, which was initially used to manufacture computer microchips [80]. Microfluid systems can be used to culture a cell monolayer, spheroids, organoids, or *ex vivo* tissue slices, both — separately and in combination [81–83]. More complex chips combine several cell and tissue types, which can be connected directly through a porous membrane covered by extracellular matrix components. Cell and tissue viability can be maintained within a long period of time (from weeks till months) due to checking micro-environment parameters and perfusion fluid flows (temperature, pH, nutrients and growth factors, mechanical signals resulting from pressure and fluid flows). Moreover, there has been demonstrated the possibility to line canals by human endothelial cells and substitute a cultural medium by whole blood in order to study endothelial activation, adhesion of platelets, formation of a fibrin clot in response to monoclonal antibody against CD40L designed to treat autoimmune disorders [84]. Currently, microfluid systems are being regularly used by pharmaceutical companies and some research groups worldwide as a tool to develop antitumor drugs, study invasion and metastasis processes [85].

Hassell et al. [86] developed an *in vitro* human non-small-cell lung cancer model in a microfluid chip, which simulated tumor growth in the micro-environment typical for the lung, and demonstrated a response to protein kinase inhibitor therapy. Earlier a response was observed only in *in vivo* studies. A chip had two additional side cameras to imitate physiologic respiratory movements due to cyclic resorption. The resorption rhythmically deformed flexible side walls and a horizontal membrane with tumor and epithelial cells. Using the functions of mechanical activation of the system revealed previously unknown resistance of lung cancer cells carrying two mutations *EGFR* (L858R and T790M) to tyrosine kinase inhibitors of the first and third generations — Erlotinib and Rociletinib. When culturing in standard static conditions, the culture exhibited high sensitivity to Rociletinib in sufficiently small concentrations (IC_50_ semi-inhibitory concentration is 1 nanomolar) and low sensitivity to Erlotinib (IC_50_ — 100 nanomolar). In mechanical movements imitating respiratory function the same culture was resistant to both drugs. The authors concluded that such resistance related to respiratory movements was likely to be mediated by the changes in signal transmission through EGFR receptor and MET protein kinase. The findings give a potential explanation of high therapy resistance of patients with minimal residual disease in the lungs, which remain functionally aerated and mobile [86].

Choi et al. [87] in their work reconstructed 3D structural organization and microenvironment of breast cancer. The authors cultured breast cancer spheroids and epithelial cells of lactiferous ducts and fibroblasts in gel, which imitated epithelial and stromal compartments. On spheroid periphery there were mixed populations of actively proliferating tumor and normal epithelial cells, however, their growth was limited by epithelial compartment, not resulting in tumor cell invasion in the underlying stroma with fibroblasts. Affected by Paclitaxel, spheroid diameter remained unchanged or slightly reduced. Such system makes an opportunity for modeling and studying structural and functional association of tumor cells with other cell types in the lactiferous duct and stromal compartment, which play a crucial role in breast cancer progressing and metastasing.

There are more complex models on chips containing *ex vivo* tissue samples. Shim et al. [83] modeled the relations between a tumor and a lymph node to test if a model of two organs on a chip would be able to reconstruct key features of tumor-induced immunosuppression. Murine lymph node slices were cultured together with tumor and healthy slices on a chip with recirculating media, and then their capability to respond to T cell stimulation was studied. In a model ‘lymph node–tumor’ lymph node slices appeared to be more immunosuppressed than those in a model ‘lymph node-healthy tissue’ prompting suggestions that it is possible to model successfully some features of tumor and immunity interaction using microfluid systems.

Cell viability in cell culture when exposed to drugs is assessed by basic standard techniques. Among these are MTT assay — a colorimetric test based on reduction of tetrazolium dye to insoluble formazan with purple staining, and a luminescent assay, which enables to assess ATP amount by luciferin-luciferase reaction behavior. These approaches require a great amount of cell material that is not always possible when working with patient-derived samples. A novel promising method to assess an early response of tumor cells to drugs has been considered recently: a metabolism analysis using fluorescent time-resolved microscopy of endogenous metabolic cofactors [88–90]. A number of studies have demonstrated metabolic changes to precede morphological manifestations of cell death under drugs [91–93], and metabolic heterogeneity at a cellular level correlates with a clinical tumor response [94].

In US there are two commercial systems to determine the drug sensitivity of tumor cells: MiCK (DiaTech Oncology) based on apoptosis detection in cells when exposed to drugs *in vitro* [95, 96], and ChemoFx (Precision Therapeutics) focused on determining the number of living cells using a nuclear stain DAPI at an endpoint [97]. Clinical findings involving these test-systems are few so far, and insufficient to be recommended for usage.

A drug sensitivity analysis of tumor cells isolated from patient’s tumor based on several assessment criteria obtained by independent methods using one and the same sample is a promising approach to solve a problem of individualization and chemotherapy efficiency improvement. But generally, the most complicated problem in estimating a cell response to a testing drug is still to isolate from a patient’s tumor the necessary amount of cells that are analyzable, and to maintain their viability within a certain period of time needed to provide treatment and develop a response to therapy.

## Conclusion

The review of current studies showed an urgent need in developing individualization methods of drug therapy and their introduction into clinical practice. It is obvious that such methods should predict a clinical response stiffly accurately and be realized at the least cost and within a reasonable period of time.

Two holistic approaches can be distinguished in the individual selection of medical oncology:

1. efficiency prognosis of chemotherapeutic agents and target agents based on a molecular and genetic tumor analysis;
2. direct testing of tumor drug sensitivity when tumor cells are exposed to an agent; tumor cells being isolated from a tumor and maintained viable under laboratory conditions (see the Figure).

**Figure:**
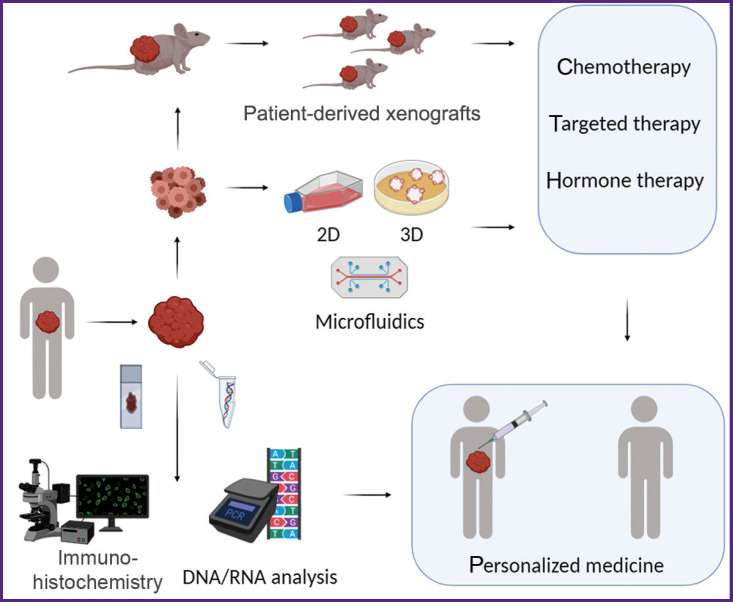
Principal approaches to testing drug sensitivity of patients’ tumors

The first approach has already proved itself when selecting agents and their combinations, the most effective regarding a particular patient tumor considering its molecular and genetic peculiarities. Moreover, known molecular mechanisms participating in tumor carcinogenesis and progressing can also be a therapeutic target for targeted therapy. The search for molecular markers reliably correlating with a therapeutic tumor response is under way now.

A promising approach to a personalized therapy is the selection of drugs on the material isolated from a tumor based on the direct assessment of a therapy effect on tumor cells. The efforts of researchers worldwide are aimed at the optimization of techniques dealing with postoperative or biopsy tumor material in order to maintain the tissue or the cells isolated from it viable as long as possible, and at the same time — maximally close model and maintain the conditions of tumor microenvironment, phenotypic and genotypic characteristics of the cells under study. Isolated tumor cells or slices maintained in cultural conditions are found the most relevant subjects for such researches.

A crucial task in the sphere of tumor drug sensitivity testing is also the search for cell response criteria. Numerous findings suggest high tumor heterogeneity by different parameters — from genetic to morphological ones that presumably determines a heterogeneous response of patients’ tumors to the same therapy. Conventional methods used to assess cell viability, e.g., MTT assay or specific staining to determine cell death or proliferation — fail to represent heterogeneity at a cellular level. A metabolic imaging technique with fluorescent time-resolved microscopy of endogenous fluorophores is considered to be a novel method to assess a heterogeneous response to therapy.

In conclusion, drug sensitivity can be most completely determined by a combined use of a molecular and genetic analysis and the direct assessment of a response of patient-derived cells on drugs included in a treatment protocol. It will enable to improve drug therapy efficiency and reduce the risk of side effects due to administering to a patient the agents, which are high-active to the tumor.
